# Long-range haplotype analysis of the malaria parasite receptor gene *ACKR1* in an East-African population

**DOI:** 10.1038/s41439-018-0024-8

**Published:** 2018-09-14

**Authors:** Qinan Yin, Kshitij Srivastava, Amha Gebremedhin, Addisalem Taye Makuria, Willy Albert Flegel

**Affiliations:** 1 Department of Transfusion Medicine, NIH Clinical Center, National Institutes of Health, Bethesda, MD USA; 2 Addis Ababa University Medical Faculty, Addis Ababa, Ethiopia; 3 U.S. Food and Drug Administration, Silver Spring, MD USA

## Abstract

The human *ACKR1* gene encodes a glycoprotein expressing the Duffy blood group antigens (Fy). The Duffy protein acts as a receptor for distinct pro-inflammatory cytokines and malaria parasites. We determined the haplotypes of the *ACKR1* gene in a population inhabiting a malaria-endemic area. We collected blood samples from 60 healthy volunteers in Ethiopia’s southwestern low-altitude tropical region. An assay was devised to amplify the *ACKR1* gene as a single amplicon and determine its genomic sequence. All haplotypes were resolved at 5178 nucleotides each, covering the coding sequence (CDS) of the *ACKR1* gene and including the 5′- and 3′-untranslated regions (UTR), intron 1, and the 5′- and 3′-flanking regions. When necessary, allele-specific PCR with nucleotide sequencing or length polymorphism analysis was applied. Among the 120 chromosomes analyzed, 18 *ACKR1* alleles were confirmed without ambiguity. We found 18 single-nucleotide polymorphisms (SNPs); only one SNP was novel. The non-coding sequences harbored 14 SNPs. No SNP, other than c.-67T>C, indicative of a non-functional allele, was detected. We described haplotypes of the *ACKR1* gene in an autochthonous East-African population and found 18 distinct *ACKR1* alleles. These long-range alleles are useful as templates to phase and analyze next-generation sequencing data, thus enhancing the reliability of clinical diagnostics.

## Malaria: Seeking resistant blood types in Ethiopia

Researchers have surveyed genetic diversity related to malaria resistance in a region of Ethiopia where malaria is endemic. Duffy antigens, a component of blood type, are encoded by the *ACKR1* gene, and individuals with the Duffy-negative blood type are resistant to malaria. Although the genes encoding resistant types have been identified, they have not been fully surveyed in malaria-endemic regions, where novel types are most likely to be found. Willy Flegel at the National Institutes of Health, Bethesda, USA, and co-workers sequenced *ACKR1* in 60 people from Gambela, Ethiopia, where malaria is endemic. They detected 18 variants, including one never before documented. Almost all (16 of 18) of the variants encoded the Duffy-negative blood type. The authors plan to compare the genetic diversity in this region with a nearby region where malaria is not endemic.

## Introduction

The human atypical chemokine receptor 1 gene (*ACKR1*, MIM #613665)^1,2^ encodes a multi-pass trans-membrane glycoprotein. It is a receptor for pro-inflammatory cytokines, such as interleukin-6 and -8^3,4^, and the malaria parasites *Plasmodium vivax* and *Plasmodium knowlesi*^5–7^. Other than its expression in erythroid cells, the ACKR1 glycoprotein (also known as Duffy) is expressed on Purkinje neurons^8^, venular endothelial cells in skin^9^, the epithelial cells of renal collecting ducts, and pulmonary alveoli^10^, and on the endothelial cells lining postcapillary venules throughout the body, except in the liver^10,11^.

The ACKR1 glycoprotein carries the five antigens of the Duffy blood group system (Fy)^12^. The two major antithetical antigens Fy^a^ and Fy^b^, encoded by the co-dominant alleles FY*A (FY*01) and FY*B (FY*02), are among the clinically most significant blood group antigens, involved in severe hemolytic transfusion reactions and hemolytic disease of the fetus and newborn^13–17^. FY*A and FY*B allele frequencies range from only 0 to 5% in East Africa^18,19^.

Fy(a-b-) is the most common phenotype in West Africans^20,21^, East Africans^22,23^, and African Americans^24^. The cause of this prevalent Duffy-null phenotype is a homozygous inheritance of a point mutation (−67T>C; rs2814778) in a regulatory element of the FY*B allele promoter^5,24–28^, found in the recessive allele FY*02N.01. This *GATA box* mutation disrupts a binding site for the GATA-1 erythroid transcription factor and abolishes the expression of the ACKR1 protein on red blood cells only. The ACKR1 protein remains expressed on cells of non-erythroid tissues^13,29^, which prevents patients from forming alloantibodies to Fy^b^ and Fy3^27^.

Despite the importance of ACKR1 in malaria infection, only one study has systematically analyzed the *ACKR1* gene at the haplotype level^30^, and no long-range *ACKR1* haplotype has been confirmed in any malaria-endemic area. The population in Gambela, a southwestern region of Ethiopia, is indigenous and has been exposed to malaria for many generations^31^. The existence of a strong selective pressure for malaria resistance implies that the Ethiopian population has a propensity to develop and maintain distinct malaria-resistant *ACKR1* haplotypes. We identified long-range haplotypes and variations of the *ACKR1* gene, including potential regulatory elements, without ambiguity, in an autochthonous population from a malaria-endemic area.

## Materials and methods

### Human research subjects

Healthy volunteers, age 18 and older, participated with informed consent in the NIH protocol NCT01282021. The blood samples from 57 individuals were collected at the Gambella Blood Bank, Gambela region, Ethiopia, and then transported to NIH. For comparison, three additional Ethiopian samples were drawn in Addis Ababa. The DNA was extracted (EZ1 DNA blood kit on a BioRobot EZ1 Workstation; Qiagen, Valencia, CA) from ethylenediaminetetraacetic acid (EDTA)-anticoagulated whole blood.

### *ACKR1* gene amplification

We devised a sequencing approach capturing the whole 2204-bp NM_002036.3 mRNA transcript, intron 1, and the 5′- and 3′-flanking regions harboring the promoter and other regulatory elements. A 12,125-nucleotide stretch of the *ACKR1* gene was amplified as a single primary amplicon from 50 ng of genomic DNA using a long-range Taq polymerase (LongAmp Taq DNA Polymerase; New England Biolabs, Ipswich, MA, USA) and the first-round primers 5′-GCATTGCTTCCAGTTCTAAGCTC-3′ and 5′-CGTCTCAATCGGTCCCTAAATCC-3′ (Eurofins MWG Operon; Huntsville, AL). The thermocycling conditions were as follows: initial denaturation at 94 °C for 2 min; 30 cycles at 94 °C for 30 s, 55 °C for 1 min, and 65 °C for 13 min; and a final extension at 65 °C for 10 min (DNA Engine Tetrad 2 Peltier Thermal Cycler; Bio-Rad, Hercules, CA).

The first-round reaction product (1 µl) was inoculated into the second-round polymerase chain reaction (PCR) using the nested primers 5′-CAACCACTCCTCCCATGGCATT-3′ and 5′-GATGAGGAGGGGTTTCTGTCC-3′ (Eurofins MWG Operon) to generate an amplicon of 5782 nucleotides. The thermocycling conditions were as follows: initial denaturation at 94 °C for 2 min; 30 cycles at 94 °C for 30 s, 62 °C for 1 min, and 65 °C for 6 min; and a final extension at 65 °C for 10 min.

### Nucleotide sequencing

The primers for sequencing were designed using Primer3 (Table S1). The nested amplicon was purified and sequenced as previously described^30^, with extensive confirmatory resequencing. The nucleotide sequences were aligned (CodonCode Aligner, CodonCode, Centerville, MA) to NCBI RefSeq NG_011626.3, and the nucleotide positions were defined using the first nucleotide of the coding sequence (CDS) of NM_002036.3. Our sequencing covered 5178 nucleotides of the *ACKR1* gene (NM_002036.3), including 1011 nucleotides of CDS, 480 nucleotides of intron, 1947 nucleotides of the 5′-UTR, 50 nucleotides of the 3′-UTR, 2101 nucleotides of the 5′-flanking region, and 589 nucleotides of the 3′-flanking region. This sequencing strategy captured all 642 variable positions listed for the 3736 nucleotides of the *ACKR1* gene in the dbSNP database^32^. It also covered 171 variable positions listed for another 1442 nucleotides of the 5′- and 3′-flanking regions of the *ACKR1* gene with potential regulatory elements.

### Fragment analysis

The nucleotide sequencing did not allow for the resolution of a short tandem repeat (STR) site in the 5′-flanking region of the *ACKR1* gene, comprising either eight or nine copies of a TG repeat. To differentiate an STR with nine TG copies from an STR with the c.-2872_-2871 TG deletion, indicative of eight TG copies, a PCR-based fragment analysis assay (Genewiz, Frederick, MD) was applied to all 60 samples (Table S1). The fragment lengths were scored using GeneMapper Software v4.1 (Applied Biosystems, Foster City, CA). A sample was considered to have nine TG repeats when the peak was observed at a fragment size of 145 bp, or eight TG repeats when the peak was observed at 143 bp.

### Physical confirmation of haplotypes (alleles)

Heterozygosity at a single site or complete homozygosity allowed for the unambiguous assignment of a haplotype as described previously^33^. Allele-specific PCR and subsequent sequencing of the PCR products were used to construct the haplotype structure in samples with more than one heterozygous site. Briefly, 34 allele-specific PCR primers were designed for the first and last heterozygous site found in the amplicons of 20 individuals (Table S1). Long-range allele-specific PCRs, nested in the secondary 5782-bp amplicon, were carried out, and all variant positions between the first and last heterozygous sites were sequenced.

### Computational phasing (predicted haplotypes)

The unphased genotype data from the 60 Ethiopian individuals and from the 2504 individuals from the 1000 Genomes Project were used as input data in the Markov chain-based haplotyper MaCH 1.0^34^ software. Due to the inherent uncertainty of computational phasing, we ran our Ethiopian genotype dataset using various MaCH program settings, evaluating several combinations of rounds and states. A stable number of predicted haplotypes were observed with 1000 and 2000 rounds and 500 to 12,000 states. Hence, the analysis in the Ethiopian and 1000 Genome datasets was performed with MaCH program settings of 2000 rounds and 500 states.

### Computational modeling of amino acid substitutions

PredictSNP was applied to predict the functional impact of non-synonymous nucleotide substitutions^35^.

### Statistical analysis

Ninety-five percent confidence intervals (CI) for allele frequencies were calculated using the Poisson distribution^36^. The observed genotype frequencies were examined for deviation from the Hardy–Weinberg equilibrium (HWE) using a goodness-of-fit *χ*^2^-test with one degree of freedom.

## Results

**Fig. 1:**
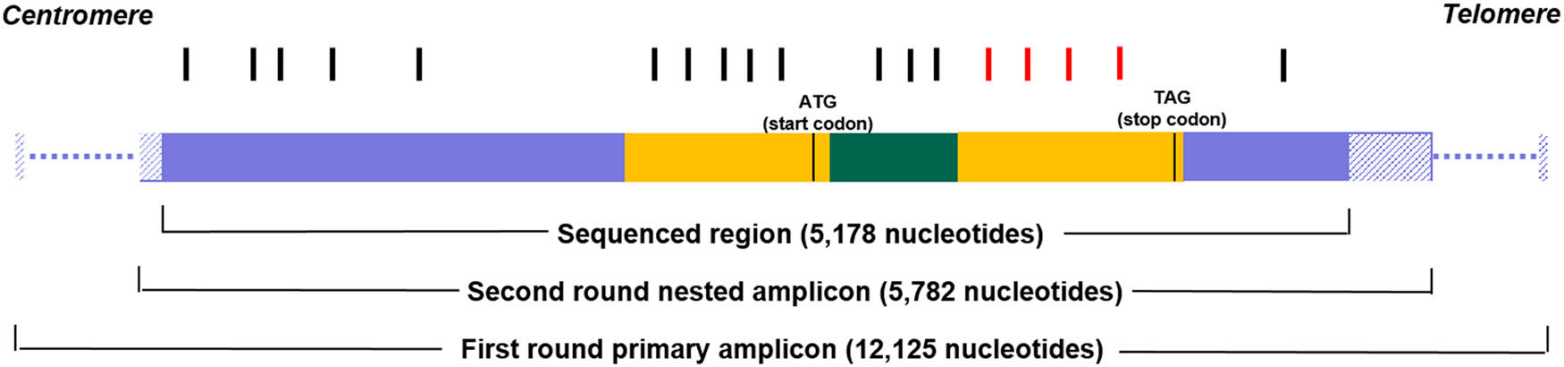
Structure of the *ACKR1* gene and SNPs found. The sequenced region included the two exons (yellow) and intron 1 (green) along with the 5′- and 3′-flanking regions (blue), covering most of the nested amplicon. The primary amplicon was much larger (dotted line, not to scale). The 18 SNPs (bars; Table S2) were located in the coding (red bar) and non-coding sequence regions (black bar)

A random survey in 60 healthy volunteers was performed to describe the genetic variability of the *ACKR1* gene for a large number of long-range haplotypes (alleles). We determined the *ACKR1* genotype for 5178 nucleotides in each individual and resolved all alleles without ambiguity (Fig. 1).

### Nucleotide variations, genotype patterns, and alleles

We observed a total of 18 nucleotide positions where single-nucleotide polymorphisms (SNPs) were present (Table 1). Except for the *GATA box* mutation, no other SNP encoding a non-sense mutation or a frame-shift mutation was found, and only one SNP in the 5′-UTR was novel and had not been previously documented in the dbSNP database. Out of the 60 individuals analyzed and a total of 310,680 nucleotides sequenced (Table S2), 20 distinct genotype patterns were observed (Table S3). Physical evidence by allele-specific amplification and sequencing allowed us to discern 18 *ACKR1* alleles (confirmed haplotypes) in the 120 chromosomes analyzed (Table 2).

**Table 1:** Genetic variations detected in the *ACKR1* gene

Location	Nucleotide change^a^	dbSNP reference no.	Protein residue change^b^	Observations (*n* = 60)				Global VAF^c^	HWE (*p*)
				Homozygote reference	Heterozygote	Homozygote variant	VAF		
5′ Flanking region	−2872_−2871TG>del	rs5778112	NA	17	24	19	0.517	0.141	0.123
	−2456T>G	rs35432289	NA	56	4	0	0.033	0.005	0.789
	−2212C>G	rs149599957	NA	59	1	0	0.008	0.003	0.948
	−1982C>T	rs34190692	NA	48	11	1	0.108	0.006	0.693
	−1310T>C	rs867811805	NA	59	1	0	0.008	<0.01	0.948
5′ UTR	−847C>T	rs114349581	NA	59	1	0	0.008	0.007	0.948
	−655A>G	rs3027011	NA	45	15	0	0.125	0.027	0.268
	−436C>T	Novel	NA	59	1	0	0.008	NA	0.948
	−399_−398CT>del	rs71782098	NA	59	1	0	0.008	0.027	0.948
	−67T>C	rs2814778	NA	0	2	58	0.983	0.266	0.896
Intron 1	+115T>C	rs7550207	NA	43	14	3	0.208	0.118	0.215
	+150C>T	rs863002	NA	59	1	0	0.008	0.201	0.948
	−243T>del	rs17838198	NA	0	0	60	1	0.222	NA
Exon 2	125G>A	rs12075	Gly42Asp	0	0	60	1	0.460	NA
	265C>T	rs34599082	Arg89Cys	59	1	0	0.008	0.005	0.948
	298G>A	rs13962	Ala100Thr	59	1	0	0.008	0.069	0.948
	602C>T	rs758176489	Thr201Met	59	1	0	0.008	0.000	0.948
3′ Flanking region	+268A>G	rs863003	NA	59	1	0	0.008	0.126	0.948

*VAF* variant allele frequency, *HWE* Hardy–Weinberg equilibrium, *NA* not applicable

^a^Nucleotide substitutions are shown relative to the reference sequence (NG_011626.3). Nucleotide positions are defined using the first nucleotide of the coding sequence (CDS) of NM_002036.3 isoform as nucleotide position 1

^b^Relative to NCBI Reference Sequence NP_002027.2

^c^Global VAF from 1000Genome, TOPMed (nhlbiwgs.org), and gnomAD (http://gnomad.broadinstitute.org/) databases

**Table 2:** *ACKR1* allele distribution in southwest Ethiopian individuals

GenBank number	Allele (confirmed haplotype)^a^	Observations (*n*)	Allele frequency (%)	
			Mean^b^	95% CI^c^
NG_011626.3	tgtcctcacctttctGCGCa	NA	NA	NA
MG932622	tgtcctcacctctc-ACGCa	36	30.0	21.2–40.6
MG932623	--tcctcacctctc-ACGCa	41	34.3	24.1–45.8
MG932624	--tcttcacctctc-ACGCa	9	7.5	3.7–14.0
MG932625	--gcctcacctctc-ACGCa	3	2.5	0.7–6.8
MG932626	--tcctcacctccc-ACGCa	2	1.7	0.3–5.6
MG932627	--tcttcacctccc-ACGCa	2	1.7	0.3–5.6
MG932628	tgtcctcacctccc-ACGCa	5	4.3	1.6–9.3
MG932629	tgtcctcgcctccc-ACGCa	12	10.0	5.6–16.9
MG932630	--gcctcatctctc-ACGCa	1	0.8	0.1–4.4
MG932631	--tgcttacctctc-ACGCa	1	0.8	0.1–4.4
MG932632	tgtccccacctctc-ACGCa	1	0.8	0.1–4.4
MG932633	tgtcctcacctttt-ATACa	1	0.8	0.1–4.4
MG932634	tgtcctcgcctccc-ACGCg	1	0.8	0.1–4.4
MG932635	tgtcctcacctttc-ACGCa	1	0.8	0.1–4.4
MG932636	--tcttcac--ccc-ACGCa	1	0.8	0.1–4.4
MG932637	--tcctcgcctccc-ACGCa	1	0.8	0.1–4.4
MG932638	tgtcctcgcctctc-ACGCa	1	0.8	0.1–4.4
MG932639	--tcttcacctctc-ACGTa	1	0.8	0.1–4.4
Total		120	100	NA

*NA* not applicable

^a^The nucleotides at the 16 SNP and two dinucleotide repeat (rs5778112 and rs71782098) positions are shown in 5′- to 3′-orientation (see Table S2). The remaining 5158 nucleotide positions that we determined had no variation relative to the reference sequence NG_011626.3. The upper case nucleotides are located in the coding sequence while the lowercase nucleotides are located in the non-coding sequence of the *ACKR1* gene

^b^Number of observed alleles × 100/Total number of alleles

^c^95% confidence interval (CI), Poisson distribution, two sided^36^

### Predicted blood group phenotype

All of the 18 alleles detected carried the variant (c.125A; p.Asp42) specific for the common Fy(b+) phenotype. The clinically relevant FY*02N.01 allele in Africans^37^, as defined only by the two SNPs at positions c.-67T>C (*GATA box* mutation) and c.125G>A (Fy^a^/Fy^b^) in the promoter and coding sequence, respectively^25^, was consistent with 16 of the 18 alleles. The other two alleles represented the reference FY*02 allele of the Fy(b+) phenotype and an FY*02W.01 allele of the Fy(b+^w^) phenotype, respectively.

### Predicted effect on protein structure

Only four non-synonymous SNPs were found (Table 3), and computational modeling by PredictSNP indicated that all four changes were neutral.

**Table 3:** Functional significance of non-synonymous SNPs predicted by PredictSNP

dbSNP reference number	Variation		Computational analysis results	
	Nucleotide change^a^	Amino acid substitution^b^	Classification	Expected accuracy (%)^c^
rs12075	c.125G>A	p.Gly42Asp	Neutral	83
rs34599082	c.265C>T	p.Arg89Cys	Neutral	74
rs13962	c.298G>A	p.Ala100Thr	Neutral	68
rs758176489	c.602C>T	p.Thr201Met	Neutral	83

^a^Relative to NCBI Reference Sequence NM_002036.3

^b^Relative to NCBI Reference Sequence NP_002027.2

^c^Normalized confidence as calculated by the software (PredictSNP)

### Predicted haplotypes by computational phasing

For comparison, we applied a common computational approach for haplotype prediction. The individual *ACKR1* haplotypes were reconstructed by running 2000 iterations (rounds) and considering 500 haplotypes (states) as the MaCH program settings (Table S4). Using our genotype information (Table S2) as input data, the MaCH software predicted 17 *ACKR1* haplotypes. Out of our 18 physically confirmed alleles, only 13 alleles (76.5%) were correctly predicted while five alleles (MG932630, MG932633 to MG932635, and MG932637) were missed (Table S5). Another four haplotypes (MaCH-01 to MaCH-04; 23.5%), which were not actually present in the 60 individuals, were predicted by MaCH as single occurrences (Table S5).

### *ACKR1* haplotypes in the 1000 Genomes project

Using the same MaCH program settings, we analyzed the *ACKR1* unphased genotype data from the 1000 Genomes Project (1000GP, phase 3)^38^. Out of the 18 SNPs detected in Ethiopia (Table 1), only 13 SNPs were found in the 1000GP database (Table S6). The MaCH software predicted 20 haplotypes using the 13 SNPs. Among our 60 Ethiopian individuals and the 2504 individuals of the 1000GP, we observed only two shared alleles (MG932622 and MG932629), which were among the most prevalent alleles in both cohorts (Table S6).

## Discussion

Previous population-based molecular studies on the malaria resistance-associated *ACKR1* gene have been centered in sub-Saharan African^22,39^ and North African Arab populations^40^. We collected blood samples from 60 individuals from Gambela, a tropical malaria-endemic region of southwestern Ethiopia, and sequenced a 5178-nucleotide region of chromosome 1 encompassing the *ACKR1* gene. This is the first study in an autochthonous African population to systematically categorize SNPs found at the *ACKR1* gene locus into long-range alleles.

The dbSNP database^32^ lists 813 nucleotide variations in the 5178-nucleotide region that we analyzed. In the present study, we observed 17 known variations and one novel variation (Table 1). Many of the variants described in the dbSNP database but not observed in our study may not be polymorphic in our population or are so rare that our screening panel lacked adequate power to detect them. No variant associated with a non-functional *ACKR1* protein was detected, besides the *GATA box* variant (rs2814778). A large fraction of the *ACKR1* alleles (>70%) occurred with low prevalence (Table 2), correlating with the known diverse genetic background of African populations^41^. The *ACKR1*-null allele FY*02N.01 was prevalent with >95%, possibly explained by the endemic *P. vivax* malaria in the region^18,31^. Non-synonymous SNPs, which introduce amino acid changes, could affect protein structure and function;^42^ however, the four such variants identified in our study had no effect on the protein structure, as calculated by PredictSNP (Table 3). Therefore, these computer predictions should be interpreted with caution because the three-dimensional structure of the complete ACKR1 protein remains unknown.

The MaCH algorithm did not identify five of the actual alleles and incorrectly predicted four haplotypes that were not present (Table S5). All of the prediction errors concerned alleles with only one observation (0.8% each). Relying on only computerized allele calling would result in 6.6% incorrect allele calls, potentially affecting one out of 15 patients (Table 4). In one individual (no. 44 in Table S5), the infrequent MG932628 allele was missed and substituted by the more frequent, albeit incorrect, allele combination MG932626 + MG932629. Thus, although computerized allele prediction may replace physical sequencing approaches for determining common alleles, our observation of incorrect predictions warns that computational algorithms can falter when rare alleles are encountered, even in this era of effortlessly obtained big data.

**Table 4:** MaCH prediction of the *ACKR1* haplotypes

Two haplotypes per individual^a^	MaCH prediction of		Rate (%)
	Haplotypes (*n*)	Individuals (*n*)	
Both correct	112	56	93.4
Both incorrect	8	4	6.6
Total	120	60	100

^a^As compared to the 18 physically confirmed alleles (MG932622 to MG932639)

The most frequent haplotype in the 1000GP samples (22.2%; Table S6) carried a T nucleotide in intron 1 (rs17838198; Table 1) that all alleles in our Ethiopian samples lacked (Table 2 and S6). This is consistent with a low prevalence of this variant in the dbSNP database for African populations (1%) compared to non-African populations (23–37%) represented in the 1000GP samples. Each of the four SNPs without frequency information in the 1000GP (Table S6, footnote) are rare and were found only once in our Ethiopian samples. The dinucleotide repeat variation rs5778112, found in 48% of our alleles, was not found in the 1000GP because variations in microsatellite regions were difficult to accurately capture^43,44^.

We experimentally verified long-range haplotypes of the *ACKR1* gene among an autochthonous Ethiopian population. As an adjunct to the human reference genome assembly (GRCh38), which is the gold standard reference^45^, our comprehensive, population-specific data and alleles are useful as template sequences for allele calling in high-throughput, next-generation sequencing and precision medicine approaches^46,47^. The design of our protocol will eventually allow us to compare populations from the Ethiopian highland and desert regions, which are not endemic for malaria, and to analyze more blood group system genes with high-throughput methods.

Web Resources

dbSNP database, Build ID: 151 (http://www.ncbi.nlm.nih.gov/SNP/)

Genome Aggregation Database (http://gnomad.broadinstitute.org/)

Hardy-Weinberg equilibrium calculator (http://www.tufts.edu/

~mcourt01/Documents/Court%20lab%20-%20HW%20calculator.xls)

MaCH, version 1.0 (http://www.sph.umich.edu/csg/abecasis/MACH/index.html)

PredictSNP, version 1.0 (http://loschmidt.chemi.muni.cz/predictsnp/)

Primer3 software, version 0.4.0 (http://bioinfo.ut.ee/primer3-0.4.0/)

TOPMed database (https://www.nhlbiwgs.org/)

Genome Aggregation Database (gnomAD; http://gnomad.broadinstitute.org/)

Ensembl genome browser (https://useast.ensembl.org/index.html)

Names for FY (ISBT 008) Blood Group Alleles (http://www.isbtweb.org/fileadmin/user_upload/files-2015/red cells/blood group allele terminology/allele tables/008 FY Alleles v3.0 140328.pdf).

## Disclaimer

The views expressed do not necessarily represent the view of the National Institutes of Health, the U.S. Food and Drug Administration, the Department of Health and Human Services, or the U.S. Federal Government.

## Electronic supplementary material

Supporting Information
