# Supplementary material for: Long-range haplotype analysis of the malaria parasite receptor gene ACKR1 in an East-African population
Source: Hum Genome Var. 2018 Sep 14;5:26. doi: 10.1038/s41439-018-0024-8 (PMC6138691; doi:10.1038/s41439-018-0024-8)
Supplement: Supplementary file 1 — Supporting Information [file 41439_2018_24_MOESM1_ESM.pdf]

Table S1. *ACKR1* primers used in the current study

Name	Sequence (5'→3')	Orientation	Use				
			Primary amplicon	Nested PCR amplicon	Nested PCR allele-specific	Sequencing	Fragment analysis
ACKR1_F1	GCATTGCTTCCAGTTCTAAGCTC	Sense	Yes	No	No	No	No
ACKR1_R1	CGTCTCAATCGGTCCCTAAATCC	Anti-sense	Yes	No	No	No	No
ACKR1_S_F1	CAACCACTCCTCCCATGGCATT	Sense	No	Yes	No	Yes	No
ACKR1_S_F2	CCTTCCCTGGATTTCCCTCCTC	Sense	No	No	No	Yes	No
ACKR1_S_F4	TTGCTGGAGAATGTTAAGACGG	Sense	No	No	No	Yes	No
ACKR1_S_F5	TGGCCAATGAAACAGTTCCAG	Sense	No	No	No	Yes	No
ACKR1_S_F6	TTTCCCAGCACCTCCCTTATCTC	Sense	No	No	No	Yes	No
ACKR1_S_F8	CTGCAGAGACCTTGTTCTCCC	Sense	No	No	No	Yes	No
ACKR1_S_F10	CCAAGCTGTTGCTGTTGTC	Sense	No	No	No	Yes	No
ACKR1_S_F11	TAGTGATTAGGACAGTGGTGGG	Sense	No	No	No	Yes	No
ACKR1_S_R1	CTCAGACCTAAGGTGCAGAAGG	Anti-sense	No	No	No	Yes	No
ACKR1_S_R3	GCAGACACTGGCAAGAAAGTC	Anti-sense	No	No	No	Yes	No
ACKR1_S_R4	CAGGAACCTCATCTCCACA	Anti-sense	No	No	No	Yes	No
ACKR1_S_R5	AGGACATACCCAGAGGAGG	Anti-sense	No	No	No	Yes	No
ACKR1_S_R6	GATGAGGAGGGGTTTCTGTCC	Anti-sense	No	Yes	No	Yes	No
ACKR1_S_R8	CTGAGGAACCTGGGAGAGATGA	Anti-sense	No	No	No	Yes	No
ACKR1_AS_F1_T	CACCACCTATTTTTTCCCATAGT	Sense	No	No	Yes	No	No
ACKR1_AS_F1_G	CACCACCTATTTTTTCCCATAGG	Sense	No	No	Yes	No	No
ACKR1_AS_F2_C	TCTAGCAGGCCTGCCCACCCC	Sense	No	No	Yes	No	No
ACKR1_AS_F2_G	TCTAGCAGGCCTGCCCACCCG	Sense	No	No	Yes	No	No
ACKR1_AS_F3_C	ACACAGAAGCCATATTTGTAC	Sense	No	No	Yes	No	No
ACKR1_AS_F3_T	ACACAGAAGCCATATTTGTAT	Sense	No	No	Yes	No	No
ACKR1_AS_F4_G	CCAAGCCCCTCACATACG	Sense	No	No	Yes	No	No
ACKR1_AS_F4_A	CCAAGCCCCTCACATACA	Sense	No	No	Yes	No	No
ACKR1_AS_R1_A	CCTTCTGTTTTTTTTGGTTTAAT	Anti-sense	No	No	Yes	No	No
ACKR1_AS_R1_G	CCTTCTGTTTTTTTTGGTTTAAC	Anti-sense	No	No	Yes	No	No

ACKR1_AS_R2_T	GCAAAGCCTTCAGCTCCA	Anti-sense	No	No	Yes	No	No
ACKR1_AS_R2_C	GCAAAGCCTTCAGCTCCG	Anti-sense	No	No	Yes	No	No
ACKR1_AS_R3_C	TGAAGGAAGAGGAAAAAAGCG	Anti-sense	No	No	Yes	No	No
ACKR1_AS_R3_T	TGAAGGAAGAGGAAAAAAGCA	Anti-sense	No	No	Yes	No	No
ACKR1_AS_R4_T	GACTTGTGAGGCAGAGATAAGA	Anti-sense	No	No	Yes	No	No
ACKR1_AS_R4_C	GACTTGTGAGGCAGAGATAAGG	Anti-sense	No	No	Yes	No	No
ACKR1_AS_R5_A	CTGTGTATCATTGTGCACATATCT	Anti-sense	No	No	Yes	No	No
ACKR1_AS_R5_G	CTGTGTATCATTGTGCACATATCC	Anti-sense	No	No	Yes	No	No
ACKR1_AS_R6_T	TGCATATGAAGTGGAATACATGA	Anti-sense	No	No	Yes	No	No
ACKR1_AS_R6_C	TGCATATGAAGTGGAATACATGG	Anti-sense	No	No	Yes	No	No
ACKR1_AS_R7_C	CTCCCTCCCACCCCCCCG	Anti-sense	No	No	Yes	No	No
ACKR1_AS_R7_T	CTCCCTCCCACCCCCCCA	Anti-sense	No	No	Yes	No	No
ACKR1_AS_R8_T	GCCTTCAACTCACTGGGTACAGA	Anti-sense	No	No	Yes	No	No
ACKR1_AS_R8_G	GCCTTCAACTCACTGGGTACAGC	Anti-sense	No	No	Yes	No	No
ACKR1_AS_R9_T	CAGCGCCTGTGCTTCCAAGA	Anti-sense	No	No	Yes	No	No
ACKR1_AS_R9_C	CAGCGCCTGTGCTTCCAAGG	Anti-sense	No	No	Yes	No	No
ACKR1_AS_R10_T	CATCGGCTGCTTCATCTCCA	Anti-sense	No	No	Yes	No	No
ACKR1_AS_R10_C	CATCGGCTGCTTCATCTCCG	Anti-sense	No	No	Yes	No	No
ACKR1_AS_R11_C	CTCCCTCCCACCCCCCCG	Anti-sense	No	No	Yes	No	No
ACKR1_AS_R11_T	CTCCCTCCCACCCCCCCA	Anti-sense	No	No	Yes	No	No
ACKR1_AS_R12_A	TGTGTATCATTGTGCACATATCT	Anti-sense	No	No	Yes	No	No
ACKR1_AS_R12_G	TGTGTATCATTGTGCACATATCC	Anti-sense	No	No	Yes	No	No
ACKR1_AS_R13_T	CTTCAACTCACTGGGTACAGA	Anti-sense	No	No	Yes	No	No
ACKR1_AS_R13_G	CTTCAACTCACTGGGTACAGC	Anti-sense	No	No	Yes	No	No
ACKR1_tg_F3_FAM	CTGTTTCCTGCCCAAACTAAGG_FAM	Sense	No	No	No	No	Yes
ACKR1_tg_R2	CCTGGCTCTTAGCCCTCCTC	Anti-sense	No	No	No	No	Yes

Table S2. *ACKR1* genotypes observed in 60 Ethiopian individuals

Individual	Genotype*																	
A1	TG/TG	T/T	C/C	C/C	T/T	C/C	A/A	C/C	CT/CT	C/C	T/T	C/C	del/del	A/A	C/C	G/G	C/C	A/A
B1	del/del	T/G	C/C	C/C	T/T	C/C	A/A	C/C	CT/CT	C/C	T/T	C/C	del/del	A/A	C/C	G/G	C/C	A/A
C1	TG/del	T/T	C/C	C/T	T/T	C/C	A/A	C/C	CT/CT	C/C	T/T	C/C	del/del	A/A	C/C	G/G	C/T	A/A
D1	TG/TG	T/T	C/C	C/C	T/T	C/C	A/A	C/C	CT/CT	C/C	T/T	C/C	del/del	A/A	C/C	G/G	C/C	A/A
E1	TG/TG	T/T	C/C	C/C	T/T	C/C	A/A	C/C	CT/CT	C/C	T/T	C/C	del/del	A/A	C/C	G/G	C/C	A/A
F1	TG/del	T/G	C/C	C/C	T/T	C/C	A/G	C/C	CT/CT	C/C	T/T	C/C	del/del	A/A	C/C	G/G	C/C	A/A
G1	TG/TG	T/T	C/C	C/C	T/T	C/C	A/A	C/C	CT/CT	C/C	T/T	C/C	del/del	A/A	C/C	G/G	C/C	A/A
H1	TG/TG	T/T	C/C	C/C	T/T	C/C	A/G	C/C	CT/CT	C/C	T/C	C/C	del/del	A/A	C/C	G/G	C/C	A/A
I1	del/del	T/T	C/C	C/T	T/T	C/C	A/A	C/C	CT/CT	C/C	T/T	C/C	del/del	A/A	C/C	G/G	C/C	A/A
A2	del/del	T/T	C/C	C/T	T/T	C/C	A/A	C/C	CT/CT	C/C	T/T	C/C	del/del	A/A	C/C	G/G	C/C	A/A
B2	TG/TG	T/T	C/C	C/C	T/T	C/C	A/A	C/C	CT/CT	C/C	T/T	C/C	del/del	A/A	C/C	G/G	C/C	A/A
C2	TG/TG	T/T	C/C	C/C	T/T	C/C	A/A	C/C	CT/CT	C/C	T/T	C/C	del/del	A/A	C/C	G/G	C/C	A/A
D2	TG/TG	T/T	C/C	C/C	T/T	C/C	A/A	C/C	CT/CT	C/C	T/T	C/C	del/del	A/A	C/C	G/G	C/C	A/A
E2	TG/del	T/T	C/C	C/T	T/T	C/C	A/A	C/C	CT/CT	C/C	T/T	C/C	del/del	A/A	C/C	G/G	C/C	A/A
F2	TG/TG	T/T	C/C	C/C	T/T	C/C	A/A	C/C	CT/CT	C/C	T/T	C/C	del/del	A/A	C/C	G/G	C/C	A/A
G2	del/del	T/T	C/C	T/T	T/T	C/C	A/A	C/C	CT/CT	C/C	T/T	C/C	del/del	A/A	C/C	G/G	C/C	A/A
H2	del/del	T/G	C/C	C/C	T/T	C/C	A/A	C/C	CT/CT	C/C	T/T	C/C	del/del	A/A	C/C	G/G	C/C	A/A
I2	TG/TG	T/T	C/C	C/C	T/T	C/C	A/A	C/C	CT/CT	C/C	T/T	C/C	del/del	A/A	C/C	G/G	C/C	A/A
A3	TG/TG	T/T	C/C	C/C	T/T	C/C	A/A	C/C	CT/CT	C/C	T/T	C/C	del/del	A/A	C/C	G/G	C/C	A/A
B3	del/del	T/T	C/C	C/T	T/T	C/C	A/A	C/C	CT/CT	C/C	T/T	C/C	del/del	A/A	C/C	G/G	C/C	A/A
C3	del/del	T/T	C/C	C/C	T/T	C/C	A/A	C/C	CT/CT	C/C	T/T	C/C	del/del	A/A	C/C	G/G	C/C	A/A
D3	TG/del	T/T	C/C	C/T	T/T	C/C	A/A	C/C	CT/del	C/C	C/C	C/C	del/del	A/A	C/C	G/G	C/C	A/A
E3	TG/TG	T/T	C/C	C/C	T/T	C/C	A/G	C/C	CT/CT	C/C	C/C	C/C	del/del	A/A	C/C	G/G	C/C	A/A
F3	TG/TG	T/T	C/C	C/C	T/T	C/C	A/A	C/C	CT/CT	C/C	T/T	C/C	del/del	A/A	C/C	G/G	C/C	A/A
G3	TG/del	T/T	C/C	C/C	T/T	C/C	A/G	C/C	CT/CT	C/C	C/C	C/C	del/del	A/A	C/C	G/G	C/C	A/A
H3	TG/TG	T/T	C/C	C/C	T/T	C/C	A/A	C/C	CT/CT	C/C	T/T	C/C	del/del	A/A	C/C	G/G	C/C	A/A
I3	TG/TG	T/T	C/C	C/C	T/T	C/C	A/A	C/C	CT/CT	C/C	T/T	C/C	del/del	A/A	C/C	G/G	C/C	A/A
A4	TG/del	T/T	C/C	C/C	T/T	C/C	A/G	C/C	CT/CT	C/C	T/C	C/C	del/del	A/A	C/C	G/G	C/C	A/A
B4	del/del	T/T	C/C	C/T	T/T	C/C	A/A	C/C	CT/CT	C/C	T/T	C/C	del/del	A/A	C/C	G/G	C/C	A/A
C4	del/del	T/G	C/C	C/C	T/T	C/C	A/A	C/T	CT/CT	C/C	T/T	C/C	del/del	A/A	C/C	G/G	C/C	A/A
D4	TG/del	T/T	C/C	C/C	T/T	C/C	A/G	C/C	CT/CT	C/C	T/C	C/C	del/del	A/A	C/C	G/G	C/C	A/A
E4	TG/del	T/T	C/C	C/C	T/T	C/C	A/G	C/C	CT/CT	C/C	T/C	C/C	del/del	A/A	C/C	G/G	C/C	A/A
F4	del/del	T/T	C/C	C/T	T/T	C/C	A/A	C/C	CT/CT	C/C	T/T	C/C	del/del	A/A	C/C	G/G	C/C	A/A
G4	TG/TG	T/T	C/C	C/C	T/T	C/C	A/A	C/C	CT/CT	C/C	T/T	C/C	del/del	A/A	C/C	G/G	C/C	A/A
H4	del/del	T/T	C/C	C/C	T/T	C/C	A/A	C/C	CT/CT	C/C	T/T	C/C	del/del	A/A	C/C	G/G	C/C	A/A
I4	TG/TG	T/T	C/C	C/C	T/T	C/C	A/G	C/C	CT/CT	T/C	C/C	C/T	del/del	A/A	C/T	G/A	C/C	A/G
A5	TG/TG	T/T	C/C	C/C	T/T	C/C	A/G	C/C	CT/CT	C/C	T/C	C/C	del/del	A/A	C/C	G/G	C/C	A/A
B5	TG/TG	T/T	C/C	C/C	T/T	C/C	A/A	C/C	CT/CT	C/C	T/T	C/C	del/del	A/A	C/C	G/G	C/C	A/A

C5	TG/del	T/T	C/C	C/C	T/T	C/C	A/G	C/C	CT/CT	C/C	T/C	C/C	del/del	A/A	C/C	G/G	C/C	A/A
D5	TG/TG	T/T	C/C	C/C	T/T	C/C	A/G	C/C	CT/CT	C/C	C/C	C/C	del/del	A/A	C/C	G/G	C/C	A/A
E5	TG/TG	T/T	C/C	C/C	T/T	C/C	A/A	C/C	CT/CT	C/C	T/T	C/C	del/del	A/A	C/C	G/G	C/C	A/A
F5	TG/TG	T/T	C/C	C/C	T/T	C/C	A/G	C/C	CT/CT	C/C	T/C	C/C	del/del	A/A	C/C	G/G	C/C	A/A
G5	TG/del	T/T	C/C	C/C	T/T	C/C	A/G	C/C	CT/CT	C/C	T/C	C/C	del/del	A/A	C/C	G/G	C/C	A/A
H5	TG/del	T/T	C/C	C/C	T/T	C/C	A/G	C/C	CT/CT	C/C	C/C	C/C	del/del	A/A	C/C	G/G	C/C	A/A
I5	del/del	T/T	C/C	C/T	T/T	C/C	A/A	C/C	CT/CT	C/C	C/C	C/C	del/del	A/A	C/C	G/G	C/C	A/A
A6	del/del	T/T	C/C	C/T	T/T	C/C	A/A	C/C	CT/CT	C/C	T/C	C/C	del/del	A/A	C/C	G/G	C/C	A/A
B6	TG/del	T/T	C/C	C/T	T/T	C/C	A/A	C/C	CT/CT	C/C	T/T	C/C	del/del	A/A	C/C	G/G	C/C	A/A
C6	TG/TG	T/T	C/C	C/C	T/T	C/C	A/G	C/C	CT/CT	C/C	C/C	C/C	del/del	A/A	C/C	G/G	C/C	A/A
D6	TG/TG	T/T	C/C	C/C	T/T	C/C	A/A	C/C	CT/CT	C/C	T/T	C/C	del/del	A/A	C/C	G/G	C/C	A/A
E6	del/del	T/T	C/C	C/C	T/T	C/C	A/A	C/C	CT/CT	C/C	T/T	C/C	del/del	A/A	C/C	G/G	C/C	A/A
F6	TG/TG	T/T	C/C	C/C	T/T	C/C	A/A	C/C	CT/CT	C/C	T/T	C/C	del/del	A/A	C/C	G/G	C/C	A/A
G6	del/del	T/T	C/C	C/C	T/T	C/C	A/A	C/C	CT/CT	C/C	T/T	C/C	del/del	A/A	C/C	G/G	C/C	A/A
H6	del/del	T/T	C/G	C/C	T/T	C/T	A/A	C/C	CT/CT	C/C	T/T	C/C	del/del	A/A	C/C	G/G	C/C	A/A
I6	TG/TG	T/T	C/C	C/C	T/T	C/C	A/A	C/C	CT/CT	C/C	T/T	C/C	del/del	A/A	C/C	G/G	C/C	A/A
A7	del/del	T/T	C/C	C/C	T/T	C/C	A/A	C/C	CT/CT	C/C	T/T	C/C	del/del	A/A	C/C	G/G	C/C	A/A
B7	del/del	T/T	C/C	C/C	T/T	C/C	A/A	C/C	CT/CT	C/C	T/T	C/C	del/del	A/A	C/C	G/G	C/C	A/A
C7	TG/TG	T/T	C/C	C/C	T/T	C/C	A/A	C/C	CT/CT	C/C	T/T	C/C	del/del	A/A	C/C	G/G	C/C	A/A
D7	TG/del	T/T	C/C	C/C	T/T	C/C	A/A	C/C	CT/CT	T/C	T/T	C/C	del/del	A/A	C/C	G/G	C/C	A/A
E7	TG/TG	T/T	C/C	C/C	T/T	C/C	A/A	C/C	CT/CT	C/C	T/T	C/C	del/del	A/A	C/C	G/G	C/C	A/A
F7	TG/TG	T/T	C/C	C/C	T/C	C/C	A/A	C/C	CT/CT	C/C	T/T	C/C	del/del	A/A	C/C	G/G	C/C	A/A

* The nucleotides at the 16 SNP and 2 dinucleotide repeat (rs5778112 and rs71782098) positions with variations are shown in 5‘to 3‘orientation (Table 1). The remaining 5,158 nucleotide positions that we determined had no variation relative to the reference sequence NG_011626.3.

Table S3. *ACKR1* genotypes observed in 60 Ethiopian individuals

Genotype number	Genotype*																		Total
NG_011626.3	TG/TG	T/T	C/C	C/C	T/T	C/C	A/A	C/C	CT/CT	T/T	T/T	C/C	T/T	G/G	C/C	G/G	C/C	A/A	NA
01	TG/del	T/T	C/C	C/C	T/T	C/C	A/A	C/C	CT/CT	C/C	T/T	C/C	del/del	A/A	C/C	G/G	C/C	A/A	27
02	del/del	T/T	C/C	C/T	T/T	C/C	A/A	C/C	CT/CT	C/C	T/T	C/C	del/del	A/A	C/C	G/G	C/C	A/A	5
03	TG/del	T/T	C/C	C/C	T/T	C/C	A/G	C/C	CT/CT	C/C	T/C	C/C	del/del	A/A	C/C	G/G	C/C	A/A	5
04	TG/TG	T/T	C/C	C/C	T/T	C/C	A/G	C/C	CT/CT	C/C	T/C	C/C	del/del	A/A	C/C	G/G	C/C	A/A	3
05	TG/TG	T/T	C/C	C/C	T/T	C/C	A/G	C/C	CT/CT	C/C	C/C	C/C	del/del	A/A	C/C	G/G	C/C	A/A	3
06	del/del	T/G	C/C	C/C	T/T	C/C	A/A	C/C	CT/CT	C/C	T/T	C/C	del/del	A/A	C/C	G/G	C/C	A/A	2
07	TG/del	T/T	C/C	C/T	T/T	C/C	A/A	C/C	CT/CT	C/C	T/T	C/C	del/del	A/A	C/C	G/G	C/C	A/A	2
08	TG/TG	T/T	C/C	C/C	T/C	C/C	A/A	C/C	CT/CT	C/C	T/T	C/C	del/del	A/A	C/C	G/G	C/C	A/A	1
09	del/del	T/T	C/C	T/T	T/T	C/C	A/A	C/C	CT/CT	C/C	T/T	C/C	del/del	A/A	C/C	G/G	C/C	A/A	1
10	TG/del	T/T	C/C	C/C	T/T	C/C	A/A	C/C	CT/CT	T/C	T/T	C/C	del/del	A/A	C/C	G/G	C/C	A/A	1
11	del/del	T/G	C/C	C/C	T/T	C/C	A/A	C/T	CT/CT	C/C	T/T	C/C	del/del	A/A	C/C	G/G	C/C	A/A	1
12	TG/del	T/G	C/C	C/C	T/T	C/C	A/G	C/C	CT/CT	C/C	T/T	C/C	del/del	A/A	C/C	G/G	C/C	A/A	1
13	del/del	T/T	C/G	C/C	T/T	C/T	A/A	C/C	CT/CT	C/C	T/T	C/C	del/del	A/A	C/C	G/G	C/C	A/A	1
14	del/del	T/T	C/C	C/T	T/T	C/C	A/A	C/C	CT/CT	C/C	T/C	C/C	del/del	A/A	C/C	G/G	C/C	A/A	1
15	del/del	T/T	C/C	C/T	T/T	C/C	A/A	C/C	CT/CT	C/C	C/C	C/C	del/del	A/A	C/C	G/G	C/C	A/A	1
16	TG/del	T/T	C/C	C/T	T/T	C/C	A/A	C/C	CT/CT	C/C	T/T	C/C	del/del	A/A	C/C	G/G	C/T	A/A	1
17	TG/del	T/T	C/C	C/C	T/T	C/C	A/G	C/C	CT/CT	C/C	C/C	C/C	del/del	A/A	C/C	G/G	C/C	A/A	1
18	TG/del	T/T	C/C	C/C	T/T	C/C	A/G	C/C	CT/CT	C/C	C/C	C/C	del/del	A/A	C/C	G/G	C/C	A/A	1
19	TG/del	T/T	C/C	C/T	T/T	C/C	A/A	C/C	CT/del	C/C	C/C	C/C	del/del	A/A	C/C	G/G	C/C	A/A	1
20	TG/TG	T/T	C/C	C/C	T/T	C/C	A/G	C/C	CT/CT	T/C	T/C	C/T	del/del	A/A	C/T	G/A	C/C	A/G	1

* The nucleotides at the 16 SNP and 2 dinucleotide repeat (rs5778112 and rs71782098) positions with variations are shown in 5'- to 3'-orientation (Table 1). The remaining 5,158 nucleotide positions that we determined had no variation relative to the reference sequence NG_011626.3.

NA-Not available

Table S4. Comparison of computer analysis with different MaCH program settings using the Ethiopian genotype data

MaCH program settings				Confirmed alleles or predicted haplotypes (n)				
				MaCH program			Physical sequencing	
Rounds	States	Flips*	Alleles in 120 chromosomes correctly predicted by MaCH (n)	Mach imputed haplotypes (total)	Haplotypes imputed by Mach but not confirmed by experiment	Alleles imputed by MaCH and confirmed by experiment	Alleles confirmed by experiment but not imputed by Mach	Alleles confirmed by experiment (total)
50	200	7.4	113	17	6	11	7	18
100	400	7.2	112	17	7	10	8	18
200	800	7.3	112	17	7	10	8	18
250	1000	7.4	113	16	5	11	7	18
1000	100	7.6	113	16	5	11	7	18
1000	500	7.6	115	17	4	13	5	18
1000	1000	7.6	115	17	4	13	5	18
1000	4000	7.6	115	17	4	13	5	18
1000	8000	7.6	115	17	4	13	5	18
1000	10000	7.6	115	17	4	13	5	18
1000	12000	7.6	115	17	4	13	5	18
2000	12000	7.7	115	17	4	13	5	18
2000	10000	7.7	115	17	4	13	5	18
2000	8000	7.7	115	17	4	13	5	18
2000	4000	7.7	115	17	4	13	5	18
2000	1000	7.7	115	17	4	13	5	18
2000	500	7.7	115	17	4	13	5	18
2000	100	7.7	115	16	3	13	5	18
2000	50	8.3	115	17	4	13	5	18

* “Flips” indicate the number of switches in haplotype phase required to convert the estimated haplotypes into the final haplotypes that are reported as analysis output.

Table S5. Comparison of alleles confirmed by allele-specific PCR and haplotypes predicted by MaCH software in the 60 Ethiopian individuals

Individual*	Allele (confirmed)			Haplotype (predicted)	
	Allele 1	Allele 2		Haplotype 1	Haplotype 2
1	MG932622	MG932622		MG932622	MG932622
2	MG932623	MG932625		MG932623	MG932625
3	MG932622	MG932639		MG932622	MG932639
4	MG932622	MG932622		MG932622	MG932622
5	MG932622	MG932622		MG932622	MG932622
6	MG932625	MG932638		MG932625	MG932638
7	MG932622	MG932622		MG932622	MG932622
8	MG932622	MG932629		MG932622	MG932629
9	MG932623	MG932624		MG932623	MG932624
10	MG932623	MG932624		MG932623	MG932624
11	MG932622	MG932622		MG932622	MG932622
12	MG932622	MG932622		MG932622	MG932622
13	MG932622	MG932622		MG932622	MG932622
14	MG932622	MG932624		MG932622	MG932624
15	MG932622	MG932622		MG932622	MG932622
16	MG932624	MG932624		MG932624	MG932624
17	MG932623	MG932625		MG932623	MG932625
18	MG932622	MG932622		MG932622	MG932622
19	MG932622	MG932622		MG932622	MG932622
20	MG932623	MG932624		MG932623	MG932624
21	MG932623	MG932623		MG932623	MG932623
22	MG932628	MG932636		MG932628	MG932636
23	MG932628	MG932629		MG932628	MG932629
24	MG932622	MG932622		MG932622	MG932622
25	MG932626	MG932629		MG932626	MG932629
26	MG932622	MG932622		MG932622	MG932622
27	MG932622	MG932622		MG932622	MG932622
28	MG932623	MG932629		MG932623	MG932629
29	MG932623	MG932624		MG932623	MG932624
30	MG932623	MG932630		MG932625	MaCH01
31	MG932623	MG932629		MG932623	MG932629
32	MG932623	MG932629		MG932623	MG932629
33	MG932623	MG932624		MG932623	MG932624
34	MG932622	MG932622		MG932622	MG932622
35	MG932623	MG932623		MG932623	MG932623
36	MG932633	MG932634		MaCH02	MaCH03

37	MG932622	MG932629		MG932622	MG932629
38	MG932622	MG932622		MG932622	MG932622
39	MG932623	MG932629		MG932623	MG932629
40	MG932628	MG932629		MG932628	MG932629
41	MG932622	MG932622		MG932622	MG932622
42	MG932622	MG932629		MG932622	MG932629
43	MG932623	MG932629		MG932623	MG932629
44	MG932628	MG932637		MG932626	MG932629
45	MG932626	MG932627		MG932626	MG932627
46	MG932623	MG932627		MG932623	MG932627
47	MG932622	MG932624		MG932622	MG932624
48	MG932628	MG932629		MG932628	MG932629
49	MG932622	MG932622		MG932622	MG932622
50	MG932623	MG932623		MG932623	MG932623
51	MG932622	MG932622		MG932622	MG932622
52	MG932623	MG932623		MG932623	MG932623
53	MG932623	MG932631		MG932623	MG932631
54	MG932622	MG932622		MG932622	MG932622
55	MG932623	MG932623		MG932623	MG932623
56	MG932623	MG932623		MG932623	MG932623
57	MG932622	MG932622		MG932622	MG932622
58	MG932623	MG932635		MG932622	MaCH04
59	MG932622	MG932622		MG932622	MG932622
60	MG932622	MG932632		MG932622	MG932632

* The samples from individuals 1 to 57 were drawn in Gambela (individual genotypes A1 to C7, see Table S2) and 58 to 60 in Addis Ababa (D7 to F7).

Table S6. Comparison of alleles confirmed by allele-specific PCR and haplotypes predicted by MaCH software

Allele/haplotype	Sequence	Alleles/haplotypes (n)			
		Ethiopian samples (n=60)			1000GP (n=2,504)
		Allele (confirmed)	Haplotype (predicted)		Haplotype (predicted)
NG_011626.3	tgtcctcacctttctGCGCa	0	0		1,114
MG932622	tgtcctcacctctc-ACGCa	49	50		1,106
MG932623	--tcctcacctctc-ACGCa	28	26		0
MG932624	--tcttcacctctc-ACGCa	9	9		0
MG932625	--gcctcacctctc-ACGCa	3	4		0
MG932626	--tcctcacctccc-ACGCa	2	3		0
MG932627	--tcttcacctccc-ACGCa	2	2		0
MG932628	tgtcctcacctccc-ACGCa	5	4		0
MG932629	tgtcctcgcctccc-ACGCa	12	13		134
MG932630	--gcctcatctctc-ACGCa	1	0		0
MG932631	--tgcttacctctc-ACGCa	1	1		0
MG932632	tgtccccacctctc-ACGCa	1	1		0
MG932633	tgtcctcacctttt-ATACa	1	0		0
MG932634	tgtcctcgcctccc-ACGCg	1	0		0
MG932635	tgtcctcacctttc-ACGCa	1	0		0
MG932636	--tcttcac--ccc-ACGCa	1	1		0
MG932637	--tcctcgcctccc-ACGCa	1	0		0
MG932638	tgtcctcgcctctc-ACGCa	1	1		0
MG932639	--tcttcacctctc-ACGTa	1	1		0
MaCH-01	--tcctcatctctc-ACGCa	0	1		0
MaCH-02	tgtcctcaccttct-ATGCg	0	1		0
MaCH-03	tgtcctcgcctccc-ACACa	0	1		0
MaCH-04	--tcctcacctttc-ACGCa	0	1		0
1000GP-01	tgtcctcaccttcc-ACGCa	0	0		447
1000GP-02	tgtcctcacctttc-GCGCa	0	0		1,098
1000GP-03	tgtcctcacctttt-ACGCg	0	0		274
1000GP-04	tgtcctcacctttt-ACACg	0	0		323
1000GP-05	tgtcctcacctttt-ATACg	0	0		22
1000GP-06	tgtcctcacctttt-ACGCa	0	0		299
1000GP-07	tgtcctcacctttt-GCGCa	0	0		87
1000GP-08	tgtcctcacctctt-ACGCa	0	0		1
1000GP-09	tgtgctcgcctccc-ACGCa	0	0		1
1000GP-10	tgtcctcaccttcc-ACGCg	0	0		8
1000GP-11	tgtcttcacctctc-ACGCa	0	0		30
1000GP-12	tgtcctcacctttc-GCGCg	0	0		1

1000GP-13	tggcctcacctctc-ACGCa	0	0		26
1000GP-14	tgtcctcacctttc-GTGCa	0	0		1
1000GP-15	tgtccttacctctc-ACGCa	0	0		21
1000GP-16	tgtgcttacctctc-ACGCa	0	0		14
1000GP-17	tgtcctcgcctctc-ACGCg	0	0		1
Total		120	120		5,008

* The nucleotides at the 16 SNP and 2 dinucleotide repeat (rs5778112 and rs71782098) positions with variations are shown in 5'- to 3'-orientation (Table 1). The remaining 5,158 nucleotide positions that we determined had no variation relative to the reference sequence NG_011626.3. The nucleotides at 3 SNP positons (rs867811805, -436C>T and rs758176489) and 2 dinucleotide repeat (rs5778112 and rs71782098) positions had no frequency data available in the 1000 genomes project and the reference nucleotides from NG_011626.3 were used in their positions. The upper case nucleotides are present in exons while the lower case nucleotides are present in the non-coding regions (see Fig. 1).
